# Supplementary material for: Natural Intra- and Interclade Human Hybrid Schistosomes in Africa with Considerations on Prevention through Vaccination
Source: Microorganisms. 2021 Jul 8;9(7):1465. doi: 10.3390/microorganisms9071465 (PMC8305539; doi:10.3390/microorganisms9071465)
Supplement: Supplementary file 1 [file microorganisms-09-01465-s001.zip › microorganisms-1287778-supplementary.pdf]

microorganisms MDPI

Supplementary Materials: Natural Intra- and Interclade Human Hybrid Schistosomes in Africa with Considerations on Prevention through Vaccination

Table S1. Frequency of mammal natural hosts by order, family and species for *Schistosoma* groups and species and *Orientobilharzia turkestanicum*.

Parasite Group (references)		*S. haematobium* [131–133]									*S. mansoni* [131–143]					*S. indicum* [144–149]					*S. japonicum* [132,150–154]						TOTAL
**Parasite Species**		*S. haematobium*	*S. bovis*	*S. curassoni*	*S. intercalatum*	*S. guineensis*	*S. leiperi*	*S. margrebowiei*	*S. mattheei*	**TOTAL**	*S. mansoni*	*S. edwardiense*	*S. hippopotami*	*S. rodhaini*	**TOTAL**	*S. indicum*	*S. incognitum*	*S. nasale*	*S. spindale*	**TOTAL**	*S. japonicum*	*S. malayensis*	*S. mekongi*	*S. sinensium*	**TOTAL**	*O. turkestanicum*	
**HOST ORDER AND FAMILY** (species see notes): **Cetartiodactyla**	Bovidae	2	12	4			20	16	16	**70**	3				**3**	3	2	5	3	**13**	4				**4**	4	**94**
	Camelidae		1							**1**																1	**2**
	Cervidae																				3				**3**		**3**
	Suidae	1	2						2	**5**							1			**1**	2				**2**	1	**9**
	Giraffidae								1	**1**																	**1**
	Hippopotamidae											1	1		**2**												**2**
**Rodentia**	Muridae	2	2						4	**8**	27			11	**38**		2		5	**7**	21	2		2	**25**		**78**
	Caviidae										1				**1**												**1**
	Cricetidae										2				**2**												**2**
	Echimyidae										1				**1**												**1**
	Hystricidae																				1				**1**		**1**
	Sciuridae																				1				**1**		**1**
**Primates**	Cercopithecidae	5							4	**9**	10				**10**						2	1			**3**		**22**
	Cebidae										1				**1**												**1**
	Hominidae	2	1		1	1		1	1	**7**	3				**3**		1			**1**	1	1	1		**3**		**14**
**Carnivora**	Canidae										1			1	**2**		1			**1**	3		1		**4**		**7**
	Felidae													1	**1**				1	**1**	3				**3**		**5**
	Herpestidae																				1				**1**		**1**
	Procyonidae										1				**1**												**1**
	Mustelidae																				3				**3**		**3**
	Viverridae																				2				**2**		**2**
**Perissodactyla**	Equidae		3				2	2	2	**9**											2				**2**	1	**12**
**Insectivora**	Soricidae										2				**2**						2				**2**		**4**
	Erinaceidae																				1				**1**		**1**
**Lagomorpha**	Leporidae																				2				**2**		**2**
**Didelmorphia**	Didelphidae										3				**3**												**3**

**Edentata**	Myrmecophagidae										1				**1**												**1**
**TOTAL**		**12**	**21**	**4**	**1**	**1**	**22**	**19**	**30**	**110**	**56**	**1**	**1**	**13**	**71**	**3**	**7**	**5**	**9**	**24**	**54**	**4**	**2**	**2**	**62**	**7**	**274**

Notes: S.=Schistosoma; O.=Orientobilharzia; **Bovidae species** (Ovis aries, Syncerus caffer, Bos spp, Bos indicus, Bos taurus, Bubalus bubalis, Capra hircus, Damaliscus lunatus korrigum, Hippotragus equinus, Kobus ellipsiprymnus defassa, Kobus kob, Redunca redunca, Sylvicapra grimmia, Tragelaphus spekii, Aepyceros melampus, Alcelephus lichtensteini, Connochaetes taurinus, Ourebia ourebi, Raphiceros sharpie, Taurotragus oryx, Tragelaphus scriptus, Tragelaphus strepsiceros, Kobus leche, Kobus vardoni, Redunca arundinum, Hippotragus niger, Kobus ellipsiprymnus defassa, Kobus ellipsiprymnus ellipsiprymnus, Tragelaphus spekii, Tragelaphus angasii); **Caviidae species** (Cavia aperea aperea); **Cervidae species** (Hydropotes inermis, Muntiacus reevesi, Cervus timorensis); **Cricetidae species** (Akodon cursor, Calomys expulsus); **Echimyidae species** (Proechimys albispinus); **Camelidae species** (Camelus dromedarius); **Suidae species** (Sus scrofa, Phacochoerus aethiopicus, Potamochoerus porcus, Sus scrofa chridontus, Sus scrofa domesticus); **Giraffidae species** (Giraffa Camelopardalis); **Hippopotamidae species** (Hippopotamus amphibius); **Muridae species** (Arvicanthis niloticu, Otomys spp, Lophuromys flavopunctatus, Praomys natalensis, Lemniscomys griselda, Otomys angoniensis, Tatera leucogaster, Bolomys lasiurus, Dasymys incomtus, Gerbillus pyramidum, Holochilus brasiliensis, Holochilus sciureus, Lemniscomys rosalia, Lemniscomys striatus, Mastomys huberti, Nectomys squamipes, Oenomys hypoxanthus, Oryzomys nigripes, Oryzomys squamipes, Oryzomys subflavus, Oryzomys utiaritensis, Oxymycterus angularis, Pelomys fallax, Rattus norvegicus, Rattus rattus, Rattus rattus frugivorus, Tatera robusta robusta, Zygodontomys brachyurus, Zygodontomys brevicauda, Malacomys longipes, Otomys irroratus, Praomys jacksoni, Praomys morio, Thallomys paedulcus, Bandicota indica, Rattus argentiventer, Rattus nitidus, Rattus rattus diardii, Rattus tiomanicus jalorensis Apodemus agrarius ningpoensis, Apodemus speciosus, Bandicota indica nemor, Bandicota indica nemor, Eothenomys melanogaster colurnus, Eothenomys smithii, Eothenomys smithii, Microtus montebelli, Mus musculus, Rattus confucianus, Rattus fulvescens, Rattus flavipectus, Rattus chrysocomus rallus, Rattus celebensis, Rattus exulans, Rattus hoffmanni, Rattus losea sakeratensis, Rattus exulans, Rattus hoffmanni, Rattus marmosurus, Rattus norvegicus caraco, Rattus muelleri, Rattus tiomanicus, Mus caroli); **Hystricidae species** (Hystrix brachyura subscritata); **Sciuridae species** (Callosciurus erythraeus); **Cercopithecidae species** (Cercopithecus mitis, Cercopithecus pygerethrus, Papio anubis, Papio ursinus, Papio ursinus, Cercopithecus albogularis, Cercopithecus aethiops aethiops, Cercopithecus aethiops sabaeus, Erythrocebus patas, Papio cynecephalus, Papio hamadryas, Papio papio, Macaca fascicularis, Macaca mulatta); **Cebidae species** (Saimiri spp); **Hominidae species** (Homo sapiens, Pan troglodytes, Pan satyrus); **Canidae species** (Canis familiaris, Nyctereutes procyonoides procyonoides, Vulpes vulpes hoole); **Felidae species** (Leptailurus serval, Panthera leo, Felis bengalensis chinensis, Felis domestica, Felis pardus fusca); **Herpestidae species** (Herpestes urva); **Procyonidae species** (Procyon carnivorus nigripes); **Mustelidae species** (Meles meles leptorhynchus, Melogale moschata ferreogrisea, Mustela sibirica davidania); **Viverridae species** (Viverricula indica pallida, Viverra tangalunga); **Equidae species** (Equus asinus, Equus caballus, Equus caballus*Equus asinus, Equus burchelli, Equus equus); **Soricidae species** (Crocidura luna, Crocidura olivieri, Crocidura attenuata, Suncus murinus); **Erinaceidae** (Erinaceus europaeus dealbatus); **Leporidae species** (Lepus europaeus, Lepus sinensis); **Didelphidae species** (Didelphis albiventris, Didelphis aurita, Lutreolina crassicaudata); **Myrmecophagidae species** (Myrmecophaga tridactyla).
